# Correction: Rearing methods and life cycle characteristics of *Chironomus* sp. *Florida* (Chironomidae: Diptera): A rapid-developing species for laboratory studies

**DOI:** 10.1371/journal.pone.0321565

**Published:** 2025-04-01

**Authors:** Roberto Reyes-Maldonado, Bruno Marie, Alonso Ramírez

The species name *Chironomus sp. Florida* appears incorrectly throughout the article. The correct species name is *Chironomus vitellinus*.
