# Supplementary material for: Rearing methods and life cycle characteristics of Chironomus sp. Florida (Chironomidae: Diptera): A rapid-developing species for laboratory studies
Source: PLoS One. 2021 Feb 22;16(2):e0247382. doi: 10.1371/journal.pone.0247382 (PMC7899323; doi:10.1371/journal.pone.0247382)
Supplement: Attachment — Submitted filename: Response to Reviewers.pdf [file pone.0247382.s002.pdf]

Instituto de Neurobiología
Universidad de Puerto Rico
Recinto Ciencias Medicas

Institute of Neurobiology
University of Puerto Rico
Medical Sciences Campus

Roberto Reyes Maldonado
Ph.D. Candidate

February 3, 2021
J. Joe Hull, Ph.D.
Academic Editor
PLOS ONE

Dear Dr. Hull,

We would like to express our gratitude for considering our manuscript for publication in PLOS ONE. We were extremely pleased with the very positive comments of the reviewers.

We have followed your recommendations as well as the reviewers' comments and have addressed them in our revised manuscript. We modified the format in the Methodology section and applied the past tense. We have also edited the text, reducing the size as recommended. Subheadings were simplified in some of the sections. All the changes were tracked with the Microsoft Word "Track Changes" option and uploaded as requested.

We trust these changes will comply with the reviewers' requests and are looking forward to your decision.

Sincerely,

Roberto Reyes Maldonado, B.Sc.
